# Effect of Pulping Waste Liquid on the Physicochemical Properties and the Prediction Model of Wheat Straw Residue Granular Fuels

**DOI:** 10.3390/polym16060848

**Published:** 2024-03-19

**Authors:** Lanxin Xue, Guihua Yang, Zhaoyun Lin, Jinze Li, Bo He, Jiachuan Chen

**Affiliations:** State Key Laboratory of Biobased Material and Green Papermaking, Qilu University of Technology (Shandong Academy of Sciences), Jinan 250353, China; xuelanxin1024@163.com (L.X.); x1062310349@163.com (J.L.); hebo123@126.com (B.H.)

**Keywords:** pulping waste liquid, bio-based granular fuel, forming process

## Abstract

Herein, wheat straw residue and pulping waste liquid were collected from pulping mill and mixed to prepare bio-based granular fuels by using compression molding technology, and to explore the comprehensive utilization of the industrial waste of pulping and papermaking. The effects of pulping waste liquid on granular fuel properties were analyzed systemically. Further study of the function of pulping waste liquid, cellulose and hemicellulose was used to replace wheat straw residue and avoid the interference factors. Therefore, the prediction models of granular fuels were established with influencing factors that included cellulose, hemicellulose and pulping waste liquid. The granular fuels had the best performance with 18.30% solid content of pulping waste liquid. The highest transverse compressive strength of granular fuel was 102.61 MPa, and the activation energy was 81.71 KJ·mol^−1^. A series of curve fitting prediction models were established to clarify the forming process of granular fuel, and it turned out that the pulping waste liquid could improve the adhesion between solid particles and increase their compression resistance.

## 1. Introduction

Biomass granular fuel, as a renewable and environmentally friendly energy, is produced and expected to replace coal, oil, electricity and natural gas [1,2]. Therefore, the development of biomass granular fuel technology could compensate the shortage of conventional energy and provide significant environmental benefits. However, the traditional granular fuels made by molding technology has low quality, and various pollutants are released during the combustion process [3,4]. Consequently, selecting suitable additives is a crucial solution to improve the quality of granular fuels and reduce power consumption during granularizing processes [5].

Several industrial wastes as additives have been reported to improve the properties of biomass granular fuel and the physical properties and combustion characteristics of biomass granular fuel. It was discovered that using coal tar residue (CTR) as a binder for biomass/lignite granular fuel significantly enhances the granular fuel’ mechanical strength and heating value [6]. With an increase in the proportion of CTR, the ignition temperature and activation energy of wheat straw granular fuel increased gradually. At the same time, the maximum burning rate and maximum weight loss percentage decreased. The bio-waste could be successfully densified into good-quality granular fuel by drum granulation using silicate-based binders, and the link between the properties of granules and the conditions of granulation process was established [7]. Meanwhile, the combustion behaviors of sludge–biomass mixed granular fuel with different sludge ratios in air atmosphere are investigated by thermogravimetric analysis [8]. Four main stages are dehydration, volatile oxidation, char burning, and burnout during the thermal decomposition of granular fuel. The reactivities of sludge–biomass granular fuels are improved by the increment of the mixing ratio of biomass. However, the above experiments have high emission pollution and ash slagging potential, so further research is needed. The co-granularization of agricultural and forestry waste using refuse-derived fuel lowered energy consumption during granularization slightly improved particle and bulk densities, which resulted in a significant increase in the breaking load and tensile strength of the granular fuel and reduced the ash content of the roasted biomass granular fuel. However, the durability, net calorific value, and energy density were impoverished as the refuse-derived fuel in the blend increased [9,10]. The effect of paper sludge was studied as an additive to the quality of granular fuels [11]. They observed that it improved the characteristic ash melting temperature of straw granular fuel and the mechanical properties of wheat straw granular fuel. In our previous work, the effect of pulping waste liquid on forming effect and performance of wheat straw granular fuel were studied [12]. The proper amount of pulping waste liquid is added to improve the relaxed density and durability of the fuel. However, the compositional variables in the selected natural biomass materials may interfere with the experimental results to a certain extent, and the forming process of industrial waste to granular fuel has not been fully explained. Therefore, a granular fuel model was prepared for this experiment to explore this problem. The forming process of the solid content of pulping waste liquid on biomass granular fuel was explored by changing the amount of pulping waste liquid.

The pulping waste liquid is constituted with lignin, hemicellulose, sugars, organic acids, inorganic salts, etc. [13,14], which can be considered to be used as binder to be incorporated with wheat straw residues and to prepare granular fuels. In this study, cellulose, hemicellulose, and pulping waste liquid were used as raw materials to prepare biomass granular fuel models, so the function of pulping waste liquid on the properties of granular fuels was explored systemically. Thereafter, the granular fuels were characterized by the thermogravimetric analysis method and the Coats-Redfern method to analyze their combustion performance. Based on the data relationship between the granular fuel and prediction models, the function of pulping waste liquid was clarified. These research results provide good guidance to their practical utilization, and will provide a theoretical basis for the further development of biomass granular fuel.

## 2. Materials and Methods

### 2.1. Experimental Materials

Wheat straws residues with a particle size of >0.22 mm were attained from a pulp and paper mill in Weifang, China. The wheat straws were pre-treated with pectinase and lipase for 4 h at 55 °C, and then transferred in digester with 4% of NaOH, liquid ratio of 1:4 at 100 °C for 20 min; cellulose was filtered for papermaking, and the pulping waste liquid was attained. Cellulose (particle size:180 μm) was purchased from Shanghai Aladdin Co., Ltd. (Shanghai, China). Hemicellulose (xylan, 85%, Corncob) was purchased from Shanghai Yuanye Bio-Technology Co. Ltd. (Shanghai, China).

### 2.2. Detection of Pulping Waste Liquid

The lignin content in the pulping waste liquid was measured according to GB/T 2677.8-1994 and 72% sulfuric acid method. An ion chromatograph (ICS-5000+, Thermo Fisher Scientific, Waltham, MA, USA) was applied to determine the sugar content in the pulping waste liquid. A revolving rheometer (ARES-G2, TA Instruments, New Castle, DE, USA) was used to determine the viscosity of the pulping waste liquid.

### 2.3. Sample Preparation

#### Preparation of Wheat Straws Granular Fuel and Granular Fuel Model

The granular fuels with pulping waste liquid were prepared as Granular Fuel-1 in Table 1: (1) The oven dry wheat straws residue and pulping waste liquid were mixed, and then dried in an oven at 60 °C for 24 h; (2) 1.00 g of the mixed materials was molded by powder compressing machine (FW-4A, Tianjin Botian Shengda Technology Development Co., Ltd., Tianjin, China) at room temperature with a pressure of 8 MPa and a diameter of 13 mm. As a control group, the granular fuels with water were prepared as Granular Fuel-2 in Table 1: the oven dry wheat straws residue and water were mixed, and then molded by powder compressing machine and marked as A, B, C, D, E.

In this material balance, the preparing parameters before drying were listed, the applied pulping waste liquid has a solid content of pulping waste liquid of 4.45%. After drying, the mass fraction of pulping waste liquid were 4.26%, 18.30%, 30.94%, 40.19% and 47.26%, which were corresponded to 1, 2, 3, 4, 5.

The granular fuel models with pulping waste liquid were prepared as follows: (1) The mixture of cellulose and hemicellulose with a mass ratio of 30.15:27.06 was prepared and corresponded to the mass ratio in wheat straw residues [9]; (2) and then the mixtures were mixed with pulping waste liquid as Granular Fuel Model-1 in Table 1, and dried in an oven at 60 °C for 24 h; (3) 1.00 g of the mixed materials was molded by powder compressing machine. The samples were marked as 1#, 2#, 3#, 4#, 5#. As a control group, the granular fuels model with water were prepared with the mixture of cellulose and hemicellulose as Granular Fuel Model-2 in Table 1; the preparing process was the same as above mentioned, and the samples were marked as A#, B#, C#, D#, E#, respectively.

### 2.4. Physicochemical Performance Testing

#### 2.4.1. Density

The cylindrical granular fuels were measured after setting 0 and 12 h using a thickness gauge. The diameter of the cylindrical granular fuels was kept at 13 mm, but their height was changed and recorded to calculate the relaxed densities of d (ρ = m/V).

#### 2.4.2. Compressive Strength

An electronic universal testing machine (WDW-10E, Jinan Wenteng Testing Instrument Co., Ltd., Jinan, China) was used to measure the transverse and longitudinal compressive strengths of granular fuel with a compressing rate of 10 mm/min. When the first crack occurred, the corresponding pressure was rendered as the maximum compression resistance.

#### 2.4.3. X-ray Micro-Tomography

The microstr uctural properties of the granular fuel model were investigated using X-ray microCT (Sky Scan-2211, Bruker, Ettlingen, DE, USA) with a non-destructive method. The scaled image pixel size of 1 μm was settled, which was sufficient to detect the large features within the granular fuel model.

### 2.5. Combustion Performance Test

#### 2.5.1. Calorific Value

Wheat straws residue granular fuel and granular fuel model were measured using an automatic calorimeter (ZDHW-5, Hebi Huifa Instrument Co., Ltd., Hebi, China) with the Oxygen bomb method, and the test unit was recorded with J/g.

#### 2.5.2. Thermogravimetric Experiments

The sample heating thermogravimetric curve (TG) and derivative thermogravimetric curve (DTG) of the samples were achieved using a TGA thermogravimetric analyzer (Rigaku TG-DTA8122 model, Japan TG-DTA Instruments, Kyoto, Japan). This test was performed under air atmosphere with a flow of 50 mL/min, and the temperature ranged from 25 °C to 800 °C with a heating rate of 10 °C/min. The TG–DTG standard definition method was used to analyze the igniting temperature T_i_ (°C), the maximum combustion rate v_max_ (%/min) and temperature T_max_ (°C); the average combustion rate v (%/min) and the burn-out temperature T_f_ (°C).

The index S of comprehensive combustion characteristic [15] can be calculated with the Equation (1):

(1) $$S=\frac{v_{\max}\cdot v}{T_{i}^{2}\cdot T_{f}}$$

where, S—the comprehensive combustion characteristics, %^2^·min^−2^·°C^−3^; v_max_—the maximum combustion rate, %/min; v—the average combustion rate, %/min; T_i_—the ignition temperature, °C; T_f_—the burn-out temperature, °C.

The index R_v_ of volatile components precipitation characteristics can be calculated with the Equation (2):

(2) $$R_{v}=\frac{v_{\max}}{T_{\max}\cdot \left(T_{\max}-{\;T}_{i}\right)}$$

where, R_v_—the volatile components precipitation characteristics, %·min^−1^·K^−2^; v_max_—the maximum combustion rate, %/min; T_max_—the temperature of maximum combustion rate, K; T_i_—the ignition temperature, K.

The fire index D_i_ [16] can be calculated with the Equation (3):

(3) $$D_{i}=\frac{v_{\max}}{T_{i}\cdot T_{\max}}$$

where, D_i_—the fire index, %·min^−1^·°C^−2^; v_max_—the maximum combustion rate, %/min; T_max_—the temperature of maximum combustion rate, °C; T_i_—the ignition temperature, °C.

In this experiment, the combustion thermogravimetric experiment was carried out under a constant heating rate. The Coats-Redfern method was used to fit the kinetic parameters of the first-stage reaction. Calculation was based on the Alenius formula (4), which reflected the relationship between the chemical reaction rate constant and temperature [17]:

(4) $$\frac{d\alpha }{\mathrm{dt}}=k\cdot f\left(\alpha \right)={\mathrm{Ae}}^{-\frac{E}{\mathrm{RT}}}\cdot {\left(1-\alpha \right)}^{n}$$

where, k—the reaction constant, min^−1^; A—the pre-exponential factor, min^−1^; E—the activation energy of reaction, J/mol; R—the universal gas constant, 8.314 J/(mol·K); T—the absolute temperature, K; α—the conversion rate of reaction process, %; n—the reaction order.

(5) $$\alpha =\frac{m_{0}-m_{t}}{m_{0}-m_{f}}\cdot 100\%$$

where, m_0_—the mass of sample at the initial moment of pyrolysis, mg; m_t_—the mass of sample at the moment t of pyrolysis, mg; m_f_—the quality of sample at the time when the solution is terminated, mg.

(6) $$\ln\left[\frac{-\ln(1-\alpha )}{T^{2}}\right]=\ln[\frac{\mathrm{AR}}{\beta E}\cdot \left(1-\frac{2\mathrm{RT}}{E}\right)]-\frac{E}{\mathrm{RT}}$$

For biomass fuels, $\left(1-\frac{2\mathrm{RT}}{E}\right)$≈1, the formula can be simplified to:

(7) $$\ln\left[\frac{-\ln(1-\alpha )}{T^{2}}\right]=\ln\frac{\mathrm{AR}}{\beta E}-\frac{E}{\mathrm{RT}}$$

Let $y=\ln\left[\frac{-\ln\left(1-\alpha \right)}{T^{2}}\right]$, $x=\frac{1}{T}$, build a model according to the first-level reaction y = ax + b, make $a=-\frac{E}{R}$, $b=\ln\frac{\mathrm{AR}}{\beta E}$, according to slope a and intercept b of the fitted curve; the activation energy and the pre-exponential factor can then be obtained.

### 2.6. The Analysis of Performance Index Equation

The performance indexes of granular fuel like S, R_v_, D_i_ and initial density, are calculated to establish equations with the following steps:

Firstly, the solid content of pulp waste liquid was set as x, and the performance index of the granular fuel model was labelled with y_m_. The x-y_m_ curve was fitted to obtain Equation (1) according to experimental data of S, R_v_, D_i_ and initial density of granular fuel model, respectively. Therefore, the relationship between the x and y_m_ can be expressed as following:

y_m_ = f(x)

The performance index of the granular fuel model was set as y_m_, the performance index of the granular fuel was labelled with y. The y_m_-y curve was fitted to obtain Equation (2) according to experimental data of S, R_v_, D_i_ and initial density of granular fuel. Therefore, the relationship between the y_m_ and y can be expressed as following:

y = g(y_m_)

Equation (1) was integrated with Equation (2) to obtain Equation (3). Therefore, the relationship between the x and y could be expressed as following:

y = g(f(x))

Finally, the prediction accuracy of the granular model was evaluated by the relative error value.

## 3. Results and Discussion

### 3.1. The Composition and Adhesion of Pulping Waste Liquid

**Figure 1:**
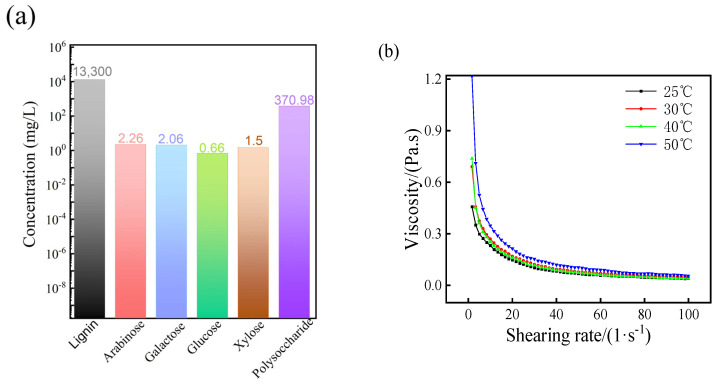
(**a**) Composition and (**b**) viscosity of pulping waste liquid.

As is shown in Figure 1a, the pulping waste liquid contains a variety of chemical components, in which lignin has the highest concentration of 13,300 mg/L followed by, polysaccharide, which has a concentration of 370.98 mg/L, and then some other sugars like arabinose, galactose, glucose and xylose, which have a concentration below 10 mg/L. In the cooking process, lignin, cellulose and hemicellulose can be hydrolyzed as small molecular carbohydrates, which have a relatively small proportion. It is known to all that lignin and sugars are natural adhesive polymers in plants, and can act as adhesive binder in molding granule. Furthermore, lignin has a high heating value of 27,000 J/g due to its rich content of carbon and hydrogen [18], so lignin can also be used to provide the quality of heat for granular fuels. So pulping waste liquid can be used to improve the performance of granular fuels.

The influence of shearing rate and temperature on pulping waste liquid are shown in Figure 1b. With the increasing shear rate, the viscosity of pulping waste liquor has a decreasing tendency, which shows a shearing–thinning behavior. Meanwhile, the viscosity of pulping waste liquid increases with the increasing temperature, the pulping waste liquid has the highest initial viscosity at 50 °C of about 5.32 Pa·s, and lowest initial viscosity at 25 °C of about 0.51 Pa·s. Lignin could dissolve in solvents at low temperature, but lignin would become soft with increasing temperature, along with the increasing viscosity. Actually, the natural adhesives like lignin, hydrolysates and other resins, waxes, are sensitive to temperature and pressure, and so they would also play binding role in the pressing process. With the increasing shearing rate, all the sample with various temperatures showed a similar viscosity of about 0.15 Pa·s, in which all the chains of the molecules have a similar directional arrangement under the shear force.

### 3.2. Effect of Pulping Waste Liquid on the Physical Properties of Wheat Straw Residue Granular Fuel

The manufactured granular fuels are influenced by many factors, like additives, moisture content, dimensions, densities, compressive strength, heating value, etc. As the important constituents and main adhesives of granular fuels, the effect of pulping waste liquid would be studied systemically.

**Figure 2:**
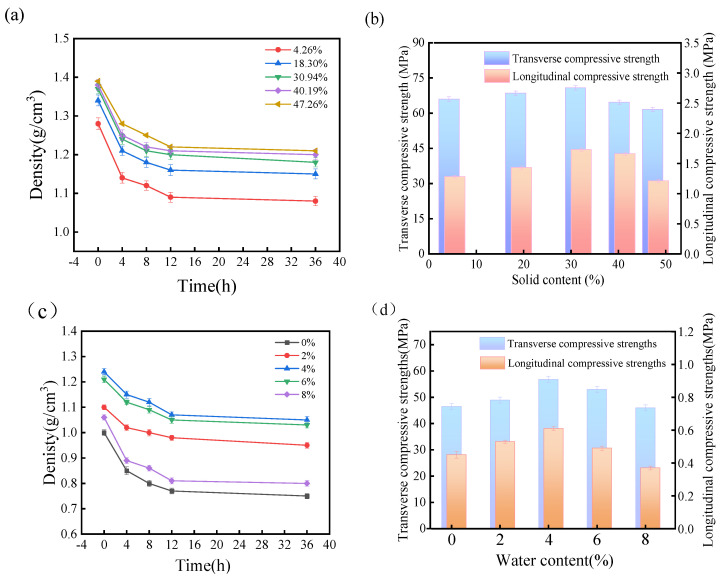
The physical properties of granular fuel with different pulping waste liquid: Density (**a**) and Compressive strength (**b**); and with water (**c**,**d**).

The relationship between the pulping waste liquid and the physical properties of granular fuel is displayed in Figure 2a. When the solid content of the pulping waste liquid increases from 4.26% to 30.94%, the density of granular fuel increases significantly, and the initial density reaches 1.37 g/cm^3^. It turns out that the solids in the pulping waste liquid can fill the gap between the wheat straw residue particles. When the solid content of waste liquid increases by 47.26%, the initial density of granular fuels decreases by 1.38 g/cm^3^, so the addition of pulping waste liquid would lead to more complicated mechanical engagement and intermolecular binding force, and finally result in a dense structure. However, the internal structure undergoes stress deformation over time, so the density of all granular fuels shows decreasing tendency with ongoing, time and the relaxation density tends to a constant after 4 h. In addition, the results show that the granular fuels with high addition of pulping waste liquid have a low reduction amplitude, and further demonstrate that the addition of pulping waste liquid improves the binding force of the granular fuels.

As shown in Figure 2b, the compressive strength of wheat straw residue granular fuel initially increases and then decreases with the increasing content of pulping waste liquid. Compared with the granular fuel with 4% water content (56.82 MPa), the best transverse compressive strength of the granular fuel increases by 9.27% with the adding of pulping waste liquid. It can be seen that pulping waste liquid has a more substantial enhancement effect on the compressive strength of straw residue granular fuel. When the solid content of pulping waste liquid is 30.94%, the transverse compressive strength reaches 62.09 MPa, which is 25.79% higher than the transverse compressive strength, with 4.26% pulping waste liquid. With the further increase of pulping waste liquid by 47.26%, the transverse compressive strength decreases to 58.40 MPa. The excessive solid content of pulping waste liquid has increased the lubricity of the internal structure [19], and thus wheat straw residue granular fuel cannot effectively support the higher external force.

Compared with the granular fuels with pulping waste liquid, the control group has low density and low physical strength and the results are listed in Figure 2c,d. Water is important in the molding process,. with increasing water proportion, water works as free radical and promotes the binding among particles, even with less external pressure. As can be seen in Figure 2c, the granular fuels have increasing transverse and longitudinal compressive strengths with increasing water content that then decreases after 4%.

### 3.3. Effect of Pulping Waste Liquid on the Physicochemical Properties of Granular Fuel Model

The granular fuels with wheat straw residue have complicate constituents, and it is hard to tell the function of pulping waste liquid clearly. Thereafter, the granular fuel models are prepared, and their physicochemical properties are analyzed and compared to clarify the forming process.

**Figure 3:**
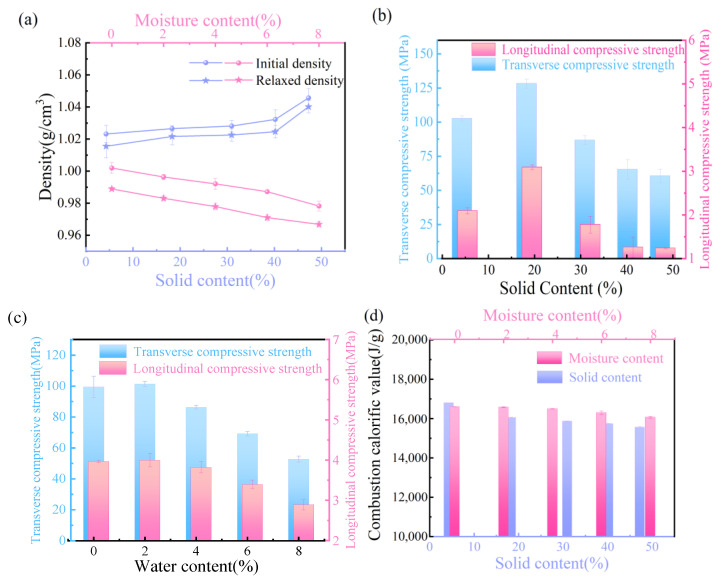
Effect of pulping waste liquor on physicochemical properties of granular fuel. (**a**) Density, (**b**,**c**) compressive strength and (**d**) combustion calorific value.

It can be seen from Figure 3a that the initial density and relaxation density show a downward tendency with the increasing moisture content. Meanwhile, the initial density and relaxation density (after 24 h) show an increasing tendency with the increasing pulping waste liquid solid content. The particle size of cellulose and hemicellulose powder is small, and their internal gap reduces without any additives. Adding water to the mixture to make the granules, the hydrogen bonding forms among cellulose, hemicellulose and water, which strengths the binding force of granular fuels. But the density decreases with increasing moisture content, and water becomes attached to the surface of particles and forms a layer of water film, which hinders the tight binding between the particles [20]. Adding the pulping waste liquid with the solid content of 4.29%, the initial density is 1.02 g/cm^3^. The initial density then increases by 1.05 g/cm^3^ with the solid content of 47.26%. Compared with the granules with water, the granules with pulping waster liquid have a smaller difference between initial and relaxed density. Lignin and sugars in the pulping waste liquid has good adhesive function [21,22] and elastic performance; they can produce plastic deformation after compression molding process, and have a fast stress transmission speed [23,24].

Figure 3b,c show that the transverse compressive strength is obviously higher than the longitudinal compressive strength of granular fuels, and they all show a descending tendency with increasing moisture. Excessive water (>2%) has a weakening effect on the compressive strength of granular fuel. However, the transverse and longitudinal compressive strength show the same tendency, which increases with increasing solid content and then decreases, and shows a maximum at 20%. The lignin and sugars in the pulping waste liquid show good bonding effect and contribute to an improved compressive strength. When the pulping waste liquid content exceeds 20%, too many spaces are filled with lignin and sugars, which creates great connectivity with cellulose and hemicellulose, and results in bad skeleton stability of the granule fuels. Therefore, the amount of pulping waste liquid should be kept at a suitable dosage.

The combustion calorific value of granular fuel model shows a downward trend with the increasing water content and pulping waste liquid solid content (Figure 3d). The pulping waste liquid containing lignin, polysaccharide etc. has a value of calorific value but is lower than cellulose/hemicellulose, which explains the reason for the decreasing tendency. Furthermore, the granular fuels with pulping waste liquid are higher than water, due to the existence of organic compounds.

### 3.4. Effect of Pulping Waste Liquid on the Combustion Characteristics of Granular Fuel Model

**Figure 4:**
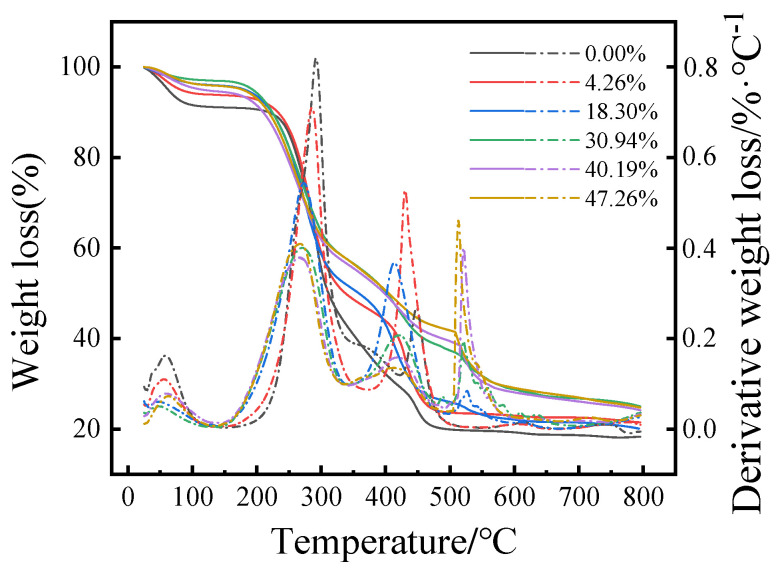
TG and DTG curves of wheat straws granular fuel with different pulping waste liquid content.

As shown in Figure 4, the evaporation temperature range of water is between 25 and 150 °C, the degradation temperature range of cellulose and hemicellulose is between 175 and 400 °C, while lignin degrades in the range from 350 to 600 °C. The higher the content of pulping waste liquid, the lower the combustion and decomposition rate of granular fuel. It is observed that the residual weight of wheat straws residue granular fuel with the most solid content (27.65%) is higher than that of wheat straws residue granular fuel without pulping waste liquor (18.96%). This is because of the presence of higher inorganic mineral content and ash in the pulping waste liquor.

**Figure 5:**
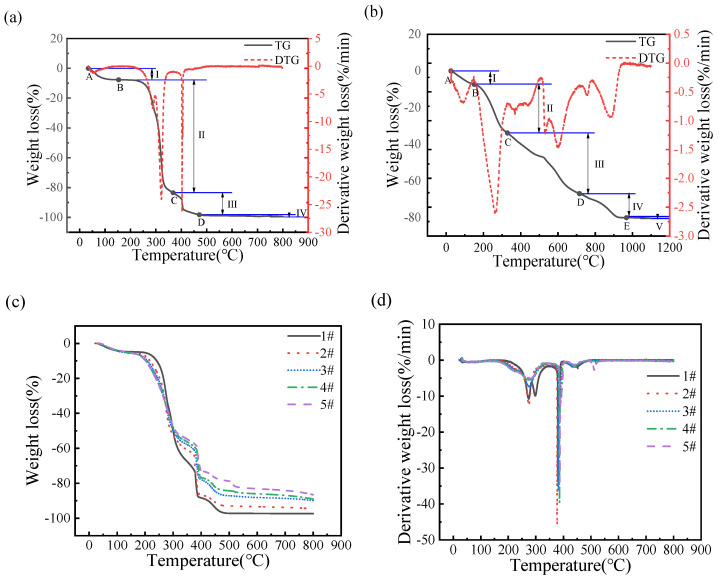
TG and DTG curves of granular fuel models with different pulping waste liquid content. (**a**) TG–DTG curves of the granular fuel model with water. (**b**) TG–DTG curves of the pulping waste liquid solids. (**c**) TG curves of granular fuel models with different pulping waste liquid content. (**d**) DTG curves of granular fuel models with different pulping waste liquid content.

It can be seen from Figure 5a that the TG curve of granular fuel mainly have four stages. They can be concluded as follows: the water evaporation stage (I), the volatile component combustion stage (II), the fixed carbon combustion stage (III) and the burn-out stage (IV); the specific data are listed in Table 2. The evaporation temperature range of water is between 25 and 150 °C, the degradation temperature of volatile components ranges from 220 to 360 °C, the fixed carbon combustion temperature ranges from 360 to 570 °C, and granular fuels burn out at 600 °C. It is known that hemicellulose pyrolysis occurs from 220 to 315 °C, and cellulose pyrolysis from 315 to 360 °C [25]. Meanwhile, CO_2_, CO and some small organic matter [26,27,28] are generated and a high weight loss of 75.45% occurs from 220 to 360 °C. At the third stage, the pyrolysis of the organic matter in the granular fuel is completed. At the last stage, the combustion is completed, and the residues are kept at 1.23%.

In referring to Figure 5b, it can be seen that the pyrolysis of pulping waste liquid mainly includes five stages. They can be concluded as follows: the water evaporation stage (I), the volatile component combustion stage (II), the fixed carbon combustion stage (III), the fourth stage (IV), and the burn-out stage (V); the detailed temperature range is listed in Table 3. At the fourth stage (480–560 °C), the oxidation and decomposition of inorganic salt in the pulping waste liquid occurs, and alkali metal salts melt, evaporate, decompose, and burn to produce volatile components such as CO and CO_2_ [29,30]. As can be seen from Table 3, with the increasing solid content of pulping waste liquid, the combustion residues of the granular fuel model also increase. The sample of A# is prepared without pulping waste liquid, and the burning residues amount of the granular fuel model is 1.23%. When the solid content of pulping waste liquid increases to 47.26%, the burning residues amount of granular fuel model increases to the maximum of 17.30%. Cellulose/hemicellulose granular fuel model with water can be completely burned, and the inorganic substances within pulping waste liquid result in residue. Therefore, when the more solid content of the pulping waste liquid is retained in the granular fuel model, more combustion residues are produced.

Figure 5c,d show the pyrolysis of granular fuel models with different pulping waste liquid content. The volatile combustion stage of granular fuel models range from 150 to 350 °C, in which the granular fuels form the initial carbon layer. With the increasing pulping waste liquid solid content, the inorganic substances proportion in the fuel model increases, followed by the melt reduction reaction recording a high initial temperature. The more inorganic substances lower the combustion temperature of the fuel model, indicating that the pulping waste liquid solid content has a synergistic effect on the combustion of the granular fuel model.

Table 3 shows that the ignition temperature of granular fuel model decreases with the increasing waste liquid solid contents. As inorganic substances are incombustible, they will decrease the ignition temperature of granular fuel model. For the granular fuel model, v_max1_ and v_max2_ show a downward trend with increasing pulping waste liquid solid content; the v of the A#, 1#, and 5# models are 4.64%·min^−1^, 3.36%·min^−1^ and 2.53%·min^−1^, respectively. The inorganic substances reduce the calorific value of the granular fuel, which results in the decrease of combustion releasing heat. Furthermore, the inorganic substances cover the unburned organic matter during the combustion process, which increases the resistance to the flame spread and decreases the combustion rate [30,31].

Table 4 shows that increasing the pulping waste liquid solid content decreases the ignition index of the granular fuel model, and weakens the ignition performance. The D_i_ of 1# fuel model is 3.91 × 10^−5^%·min^−1^·°C^−2^, and it has relatively good ignition performance; the D_i_ of 5# fuel model is 1.89 × 10^−5^%·min^−1^·°C ^−2^ with the worst ignition performance. The comprehensive combustion characteristic index of the 1# fuel model is 1.69 × 10^−7^%^2^·min^−2^·°C^−3^. The S of granular fuel decreases as the pulping waste liquid solid content increases. The R_v_ of the 1# and 5# fuel models are 2.12 × 10^−4^ and 0.65 × 10^−4^%·min^−1^·K^−2^, respectively. Therefore, the increasing waste liquid solid contents result in decreasing R_v_ of granular fuel, and a decreasing rate of the volatile components precipitation and combustion.

The kinetic parameters of granular fuel model are obtained by the first-order reaction equation in each combustion stage, and the correlation coefficients are all above 0.95, indicating that the fitting results are feasible. The activation energy E and frequency factor A of granular fuel model are calculated by fitting the slope and intercept of linear equation in each combustion stage. Herein, E is the difference between the activated molecules’ average energy and all molecules’ energy in the combustion process. The combustion process of the granular fuel is easier with lower value of E. A is the frequency factor, which is a constant determined only by the chemical substance and used to measure the intensity of the combustion process of granular fuel.

Table 5 shows that the E of the A# granular fuel model in the volatile and fixed carbon combustion stages are 124.70 and 283.99 KJ·mol^−1^, which are 136.35% and 561.67% higher than that of the waste liquid solid. The A of the A# granular fuel model in the volatile and fixed carbon combustion stages are 4.25 × 10^10^ and 3.69 × 10^21^ min^−1^, which are 1.41 × 10^6^ and 7.62 × 10^19^ times that of the waste liquid solid. Thus, under the same conditions, the volatile and fixed carbon in the waste liquid solid are more flammable than those in the A# granular fuel model, and the combustion process is milder and less prone to deflagration. The E of the 5# granular fuel model in the volatile and fixed carbon combustion stages are 55.42 and 216.86 KJ·mol^−1^, which are 55.56% and 23.64% lower than those of the A# model. This is because of the catalytic role of metal oxides in the pyrolysis process [32,33]. As the waste liquid solid content increases, metal oxides’ content in the granular fuel model increase, the catalytic pyrolysis is strengthened, the energy required for the reaction decreases, and the activation energy decreases. The A of A# granular fuel model in the volatile and fixed carbon combustion stages are 9.79 × 10^5^ times and 1.24 × 10^5^ times that of the 5# granular fuel model. This indicates that the volatile and fixed carbon of the granular fuel combined with waste liquid is more flammable, more stable, and less prone to deflagration than those without the addition of waste liquid.

### 3.5. Analysis of the Molding Process of Granular Fuel

**Figure 6:**
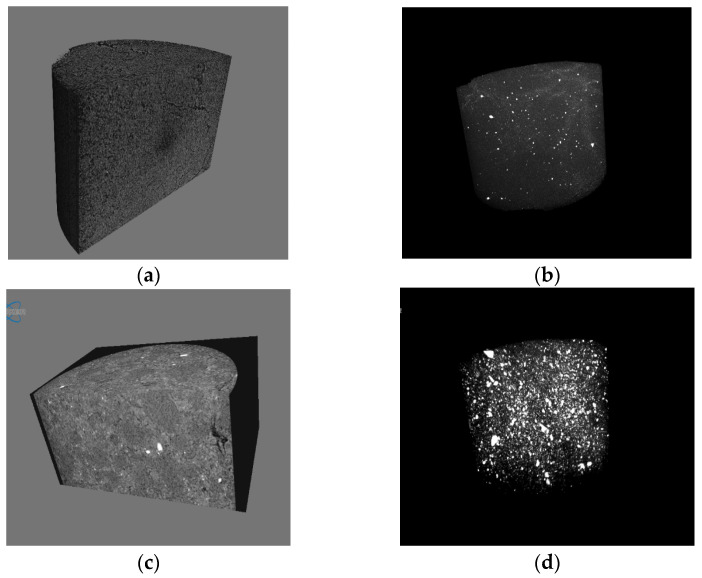
3D images of micro-CT reconstructed granular fuel model with (**a**) 2% moisture content and (**c**) 18.30% pulping waste liquid solid content; Distribution of water (**b**) and pulping waste liquid solids (**d**) in the granular fuel model.

Figure 6 shows the 3D images of the internal and external microstructure of granular fuel. The granular fuel with 2% moisture shows a regular section with small pores and cracks, and the distribution of water is shown in Figure 6b. In comparison, the granular fuel with 18.30% pulping waste liquid has a denser structure, and the liquid is closely distributed with the solid particles. It is concluded that the pulping waste liquid makes a good fusion between particles, and a strengthened binding force is formed. This can be used to directly explain the strengthened physical properties and deduced density obtained with pulping waste liquid.

The granular fuel and granular fuel model with pulping waste liquid solid content of 4.26%, 18.30%, 30.94, 40.19%, 47.26% were calculated to obtain prediction models by curve fitting and equation simplifying, with their S, R_v_, D_i_ and density listed in the following:

(8) $$y=4.06\times 10^{-7}-1.84\times 10^{-7}\cdot {\left(0.03\right)}^{e^{-\frac{x}{2.8}}}$$

In Equation (8), x refers to solid content of pulping waste liquid, and y refers to S. As is shown in Table 6, the relative error between measured data and predicated data are lower than 0.20, which means a high fitting degree exists. And then the derivation was calculated:

(9) $$\frac{\mathrm{dy}}{\mathrm{dx}}=-1.84{\times 10}^{-7}\cdot {\left(0.03\right)}^{e^{-\frac{x}{2.8}}}\cdot \ln(0.03{)\cdot e}^{-\frac{x}{2.8}}\cdot (-\frac{1}{2.8})$$

(10) $$y^{\prime }=-\frac{1{.84\times 10}^{-7}\cdot \ln\left(0.03\right)\cdot e^{\frac{x}{2.8}}}{2.8}$$

in which, x ∈ [0, 50], and y′ < 0. So, the value of S is negatively associated with the solid content of pulping waste liquid.

(11) $$y=0.28\times {\left(3.41{\times 10}^{-4}-6.00{\times 10}^{-5}\cdot x^{0.39}\right)}^{0.81}$$

In Equation (11), x refers to solid content of pulping waste liquid and y refers to R_v_. As is shown in Table 7, the relative error between measured data and predicated data are also lower than 0.20, which means a high fitting degree exists. And then the derivation was calculated:

(12) $$\frac{\mathrm{dy}}{\mathrm{dx}}=0.81\cdot 0.28\cdot {\left(3.41\times 10^{-4}-6.00\times 10^{-5}\cdot x^{0.39}\right)}^{-0.19}\cdot (-6.00\times 10^{-5})\cdot 0.39\cdot x^{-0.61}$$

(13) $$\frac{\mathrm{dy}}{\mathrm{dx}}=-[1.88{\times 10}^{5}{\cdot x}^{0.61}\cdot (3.41{\times 10}^{-4}{\;-6\;\times 10}^{-5}{\cdot x}^{0.39}{)}^{0.19}{]}^{-1}$$

(14) $$y^{\prime }=-\frac{5.31{\times 10}^{-6}}{{\left(3.41{\times 10}^{-4}-6.00{\times 10}^{-5}\cdot x^{0.39}\right)}^{0.19}\cdot x^{0.61}}$$

in which, x ∈ [0, 50] and (3.41 × 10^−4^ − 6.00 × 10 − 5∙x^0.39^) > 0, so y′ < 0. So, the value of R_v_ is negatively associated with the solid content of pulping waste liquid.

(15) $$y=0.0042\cdot {\left(7.03{\times 10}^{-5}-1.47{\times 10}^{-5}\cdot x^{0.32}\right)}^{0.38}$$

In Equation (15), x refers to solid content of pulping waste liquid and y refers to D_i_. As is shown in Table 8, the relative error between measured data and predicated data are also lower than 0.20, which means a high fitting degree exists. And then the derivation was calculated:

(16) $$\frac{\mathrm{dy}}{\mathrm{dx}}=0.0042\cdot 0.38\cdot {\left(7.03{\times 10}^{-5}-1.47{\times 10}^{-5}\cdot x^{0.32}\right)}^{-0.62}\cdot (-1.47{\times 10}^{-5})\cdot 0.32\cdot x^{-0.68}$$

(17) $$y^{\prime }=-\frac{7.51{\times 10}^{-9}}{{\left(7.03{\times 10}^{-5}-1.47{\times 10}^{-5}\cdot x^{0.32}\right)}^{0.62}\cdot x^{0.68}}$$

in which, $x\in \left[0,\;50\right]\;\mathrm{and}\;{\left(7.03{\times 10}^{-5}-1.47{\times 10}^{-5}\cdot x^{0.32}\right)}^{0.62}>0$, and $y^{\prime }<0$. So, the value of D_i_ is negatively associated with the solid content of pulping waste liquid.

(18) $$y=\frac{7.34\cdot e^{\frac{x}{7.92}}+552.62}{5.22\cdot e^{\frac{x}{7.92}}+417.17}$$

In Equation (18), x refers to solid content of pulping waste liquid, and y refers to initial density. As is shown in Table 9, the relative error between measured data and predicated data are also lower than 0.20 with the range of solid content at 4.26–47.26%. With the high fitting degree of the equation, its derivation was calculated:

(19) $$\frac{\mathrm{dy}}{\mathrm{dx}}=\frac{\left(\left(5.22{\cdot e}^{\frac{X}{7.92}\;}+417.17\right)\times 0.927{\cdot e}^{\frac{X}{7.92}\;}-\left(7.34{\cdot e}^{\frac{X}{7.92}}+552.62\right)\times 0.659{\cdot e}^{\frac{X}{7.92}\;}\right)}{{\left(5.52{\cdot e}^{\frac{X}{7.92}\;}+417.17\right)}^{2}}$$

(20) $$y^{\prime }=\frac{\left(177.35\right)\cdot e^{\frac{x}{7.92}}}{215.81\cdot e^{\frac{2x}{7.92}}+34493.62\cdot e^{\frac{x}{7.92}}+1378324.01}$$

in which, $x\in \left[0,\;50\right]$ and $y^{\prime }>\;0$. So, the value of initial density is positively associated with the solid content of pulping waste liquid.

(21) $$y=1.19-0.24\cdot e^{-\frac{x}{6.90}}$$

In Equation (21), x refers to solid content of pulping waste liquid and y refers to relaxed density. As is shown in Table 10, the relative error between measured data and predicated data are also lower than 0.20, and the equation has high fitting degree. And then the derivation was calculated:

(22) $$\frac{\mathrm{dy}}{\mathrm{dx}}=\left(-0.24\right)\cdot (-\frac{1}{6.90}){\cdot e}^{-\frac{x}{6.90}}$$

(23) $$y^{\prime }=3.48\times 10^{-2}\cdot e^{-\frac{x}{6.90}}$$

in which, x ∈ [0, 50], and y′ > 0. So, the value of relaxed density is positively associated with the solid content of pulping waste liquid.

According to the above data, the initial density and relaxed density are positively associated with the solid content of pulping waste liquid, whereas, the S, R_v_, D_i_ are all negatively associated with the solid content of pulping waste liquid. Therefore, the addition of pulping waste liquid increased the tightness and physical performance of the granular fuels, but decreased the combustion. Combined with the characteristic of pulping waste liquid and the structure of granular fuels, this confirmed the binding function of pulping waste liquid; Meanwhile, within the same quantity, pulping waste liquid has inferior combustion performance than wheat straw resides and reduced the whole combustion performance.

## 4. Conclusions

In this study, the function of pulping waste liquid on the forming process and properties of granular fuel were investigated systemically.

(1) Lignin and sugars in the pulping waste liquid were natural adhesives, and the addition of pulping waste liquid could improve the mechanical strength of granular fuels. The granular fuels had the best performance with 18.30% solid content of pulping waste liquid, the highest transverse compressive strength was up to 102.61 MPa, and the activation energy was 81.71 KJ·mol^−1^ at the volatile carbon combustion stage.
(2) The predicated models were established to study the forming process, and the pulping waste liquid were evenly distributed in granular fuels. The addition of pulping waste liquid improved their density and density combustion indexes.

## Figures and Tables

**Table 1:** The material balance of granular fuels.

Granular Fuel-1	Sample	1	2	3	4	5
	Wheat Straws Residue/g	1	1	1	1	1
	Pulping Waste Liquid/g	1	5	10	15	20
Granular Fuel Model-1	Sample	1#	2#	3#	4#	5#
	Mixture of Cellulose and Hemicellulose/g	1	1	1	1	1
	Pulping Waste Liquid/g	1	5	10	15	20
Granular Fuel-2	Sample	A	B	C	D	E
	Wheat Straws Residue/g	1	1	1	1	1
	Moisture Content/%	0	2	4	6	8
Granular Fuel Model-2	Sample	A#	B#	C#	D#	E#
	Mixture of Cellulose and Hemicellulose/g	1	1	1	1	1
	Moisture Content/%	0	2	4	6	8

**Table 2:** The combustion process analysis of the model without adding waste liquid fuel or solid content of waste liquid; granular fuel model with different solid content of the waste liquid.

Sample	Water Evaporation Stage/°C	Volatile Component Combustion Stage/°C	Fixed Carbon Oxidation Stage/°C	Fourth Stage/°C	Burnout Period/°C	Burning Remaining Amount/%
A#	–204	204–367	367–511		511–800	1.23
Waste liquid solids	–149	149–327	327–717	717–955	955–1200	19.99
1#	–177	177–347	347–520		520–800	3.15
2#	–158	158–350	350–558		558–800	6.80
3#	–150	150–343	343–555		555–800	6.82
4#	–150	150–342	342–494	494–552	552–800	14.38
5#	–138	138–340	340–483	483–557	557–800	17.30

**Table 3:** Analysis table of the thermodynamic parameters of the volatile stage combustion.

Sample	T_i_/°C	v_max1_/(%/min)	T_max1_/°C	v_max2_/(%/min)	T_max2_/°C	T_f_/°C	$\bar{v}$/(%/min)
A#	298	24.04	321	26.43	403	511	4.64
Waste liquid solids	204	2.63	262	1.54	601	955	1.53
1#	251	11.20	274	33.22	380	520	3.36
2#	249	12.44	275	46.17	377	558	2.91
3#	227	7.45	277	36.31	382	555	2.75
4#	221	5.53	278	39.71	386	552	2.64
5#	216	5.12	281	33.82	387	557	2.53

T_i_: Ignition temperature; T_f_: Burnout temperature; T_max1_: Maximum burning temperature in the volatile combustion stage; T_max2_: Maximum burning temperature in the fixed carbon stage; v_max1_: Maximum burning rate in the volatile combustion stage; v_max2_: Maximum burning rate in the fixed carbon stage; v: Average burning rate.

**Table 4:** Analysis table of the combustion characteristics of the granular fuel model.

Sample	S/%^2^·min^−2^·°C^−3^	R_v_/%·min^−1^·K^−2^	D_i_ /%·min^−1^·°C^−2^
A#	4.36 × 10^−7^	3.45 × 10^−4^	7.07 × 10^−5^
Waste liquid solids	0.14 × 10^−7^	0.43 × 10^−4^	1.02 × 10^−5^
1#	1.69 × 10^−7^	2.12 × 10^−4^	3.91 × 10^−5^
2#	1.57 × 10^−7^	1.93 × 10^−4^	4.33 × 10^−5^
3#	0.95 × 10^−7^	1.06 × 10^−4^	2.68 × 10^−5^
4#	0.70 × 10^−7^	0.79 × 10^−4^	2.03 × 10^−5^
5#	0.66 × 10^−7^	0.65 × 10^−4^	1.89 × 10^−5^

S: The comprehensive combustion characteristic index; R_v_: The volatile component precipitation characteristic index; D_i_: The ignition index.

**Table 5:** Combustion kinetic parameters of the granular fuel model.

Sample	Temperature Interval/°C	Fitting Equation	Correlation Coefficient R^2^	Activation Energy E/KJ·mol^−1^	Frequency Factor A/min^−1^
A#	220–310	y = −14999.15x + 12.55	0.9969	124.70	4.25 × 10^10^
	375–420	y = −34157.40x + 36.92	0.9613	283.99	3.69 × 10^21^
Waste liquid solids	210–300	y = −6345.99x − 0.75	0.9998	52.76	3.01 × 10^4^
	380–425	y = −5161.84x − 6.97	0.9931	42.92	4.84 × 10^1^
	790–880	y = −17846.32 + 1.55	0.9855	148.37	8.43 × 10^5^
1#	210–300	y = −12898.62x + 10.15	0.9978	107.24	3.30 × 10^9^
	360–405	y = −28286.50x + 29.02	0.9681	235.17	1.13 × 10^18^
2#	210–300	y = −9827.87x + 5.13	0.9915	81.71	1.66 × 10^7^
	355–400	y = −47576.01x + 58.70	0.9549	395.55	1.48 × 10^31^
3#	210–300	y = −8237.74x + 2.31	0.9965	68.49	8.30 × 10^5^
	365–410	y = −42527.43x + 51.22	0.9594	353.57	7.49 × 10^27^
4#	210–300	y = −6957.58x + 0.03	0.9979	57.85	7.20 × 10^4^
	365–410	y = −30575.91x + 32.41	0.9544	254.21	3.64 × 10^19^
	500–545	y = −54352.30x + 54.38	0.9647	451.89	2.25 × 10^29^
5#	210–300	y = −6666.24x − 0.43	0.9976	55.42	4.34 × 10^4^
	355–410	y = −26084.06x + 25.46	0.95367	216.86	2.97 × 10^16^
	500–525	y = −77360.42x + 84.46	0.9641	643.17	3.72 × 10^42^

**Table 6:** Prediction models of the comprehensive combustion characteristic index.

	Solid Content/%	Measured Data	Predicted Data	Relatived Error
S	0	4.01 × 10^−7^	4.01 × 10^−7^	0
	4.26	3.21 × 10^−7^	3.22 × 10^−7^	0.0031
	18.3	2.72 × 10^−7^	2.23 × 10^−7^	0.1801
	30.94	2.12 × 10^−7^	2.22 × 10^−7^	0.0472
	40.19	1.98 × 10^−7^	2.22 × 10^−7^	0.1212
	47.26	2.06 × 10^−7^	2.22 × 10^−7^	0.0777

**Table 7:** Prediction models of the volatile component precipitation characteristic index.

	Solid Content/%	Measured Data	Predicted Data	Relatived Error
R_v_	0	4.13 × 10^−4^	4.17 × 10^−4^	0.0097
	4.26	3.48 × 10^−4^	2.87 × 10^−4^	0.1753
	18.3	2.16 × 10^−4^	2.19 × 10^−4^	0.0138
	30.94	1.49 × 10^−4^	1.68 × 10^−4^	0.1275
	40.19	1.21 × 10^−4^	1.37 × 10^−4^	0.1322
	47.26	1.38 × 10^−4^	1.15 × 10^−4^	0.1667

**Table 8:** Prediction models of the ignition index.

	Solid Content/%	Measured Data	Predicted Data	Relatived Error
D_i_	0	10.99 × 10^−5^	11.11 × 10^−5^	0.0109
	4.26	9.99 × 10^−5^	9.53 × 10^−5^	0.0460
	18.3	8.69 × 10^−5^	8.35 × 10^−5^	0.0391
	30.94	6.91 × 10^−5^	7.65 × 10^−5^	0.1071
	40.19	6.83 × 10^−5^	7.21 × 10^−5^	0.0556
	47.26	7.26 × 10^−5^	6.89 × 10^−5^	0.0610

**Table 9:** Prediction models of the initial density.

	Solid Content/%	Measured Data	Predicted Data	Relativized Error
Initial Density	0	1.10	1.33	0.2091
	4.26	1.28	1.33	0.0391
	18.3	1.34	1.33	0.0075
	30.94	1.37	1.36	0.0071
	40.19	1.38	1.38	0
	47.26	1.39	1.39	0

**Table 10:** Prediction models of the relaxed density.

	Solid Content/%	Measured Data	Predicted Data	Relatived Error
Relaxed Density	0	0.95	0.95	0
	4.26	1.08	1.06	0.0185
	18.3	1.15	1.17	0.0174
	30.94	1.18	1.19	0.0085
	40.19	1.2	1.19	0.0083
	47.26	1.21	1.20	0.0083

## Data Availability

All data are contained within the article.
